# Filling the Gap between Research and Market: Portable Architecture for an Intelligent Autonomous Wheelchair

**DOI:** 10.3390/ijerph20021243

**Published:** 2023-01-10

**Authors:** Juan Carlos García, Marta Marrón-Romera, Alessandro Melino, Cristina Losada-Gutiérrez, José Manuel Rodríguez, Albert Fazakas

**Affiliations:** GEINTRA, Electronics Department, Polytechnics School, Campus Universitario, 28805 Alcalá de Henares, Spain

**Keywords:** intelligent wheelchair, node structure, CAN bus, indoor navigation, robotics, multisensors

## Abstract

Under the umbrella of assistive technologies research, a lot of different platforms have appeared since the 1980s, trying to improve the independence of people with severe mobility problems. Those works followed the same path coming from the field of robotics trying to reach users’ needs. Nevertheless, those approaches rarely arrived on the market, due to their specificity and price. This paper presents a new prototype of an intelligent wheelchair (IW) that tries to fill the gap between research labs and market. In order to achieve such a goal, the proposed solution balances the criteria of performance and cost by using low-cost hardware and open software standards in mobile robots combined together within a modular architecture, which can be easily adapted to different profiles of a wide range of potential users. The basic building block consists of a mechanical chassis with two electric motors and a low-level electronic control system; driven by a joystick, this platform behaves similar to a standard electrical wheelchair. However, the underlying structure of the system includes several independent but connected nodes that form a distributed and scalable architecture that allows its adaptability, by adding new modules, to tackle autonomous navigation. The communication among the system nodes is based on the controller area network (CAN) specification, an extended standard in industrial fields that have a wide range of low-cost devices and tools. The system was tested and evaluated in indoor environments and by final users in order to ensure its usability, robustness, and reliability; it also demonstrated its functionality when navigating through buildings, corridors, and offices. The portability of the solution proposed is also shown by presenting the results on two different platforms: one for kids and another one for adults, based on different commercial mechanical platforms.

## 1. Introduction

Nowadays, using robotics and electronic technologies to enhance the quality of life of human beings is definitely settled within our societies. From such a point of view, service robotics have gained strength in many research lines, thanks also to the improvement of electronics technologies in terms of cost, processing capacity, and low power needs.

Following such guidelines, the main objective of the system presented in this paper is to improve the independence of people with severe mobility difficulties by designing and testing appropriate autonomous wheelchair platforms, which could be affordable for final users in terms of performance, cost, and reliability.

This paper shows the result of a long trajectory [1,2,3,4] of developing assisted mobility platforms to test low-cost, open-source, and high-tech devices and solutions. As a result, two different prototypes were developed and adapted to two different profiles of potential autonomous wheelchair users. One of them is a platform adapted to kids, called KATE (Kids Assistive TEchnology); the second one is designed for adults, and is named SARA (Sistema de Ayuda Robotizado y Avanzado, in Spanish, which could be translated to Advanced Robotic Assistive System).

KATE is an intelligent wheelchair (IW) whose base is a commercial children’s car seat mounted on a mechanical chassis [5], designed ad hoc by the Padrino Tecnológico initiative [6]. SARA, was developed using a commercial powered wheelchair, following previous works [3,7].

**Figure 1:**
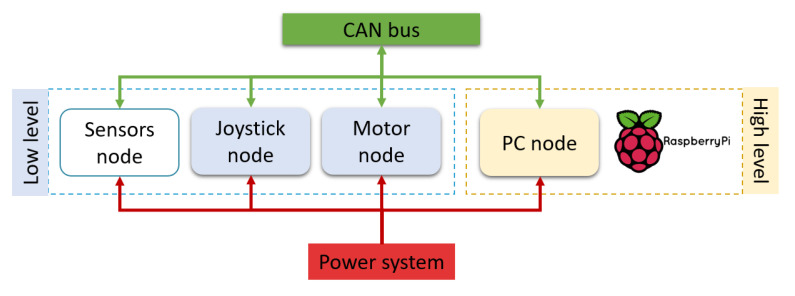
Simplified diagram of the nodal structure of the intelligent wheelchair prototypes.

As a result of the modular and standard concepts proposed, both prototypes (whose structure is shown in Figure 1) can be built using the same hardware and software modules. This approach allows easily attaching and detaching modules and behaviors (close to a plug-and-play solution), resulting in two functional incremental proposals that can always be expanded or adapted quickly to new research advances. Flexibility and scalability are important design factors in order to reduce the cost of construction, maintenance, and adaptation to users, even in the event of the progression of symptoms or illness, because the same platform could be upgraded as required by changing circumstances.

The distributed control system is based on independent nodes interconnected through a CAN bus, as shown in Figure 1. The nodes shown in such a structure are classified into two levels, according to their functions and processing power: low-level (sensors, motor and joystick) and high-level (PC or similar, i.e., a Raspberry PI). The number and devices used in the system nodes may vary according to the specific application required.

Low-level nodes comprise the basic system (in blue, in Figure 1), manually driven with different interfaces within the joystick node. High-level nodes build up the advanced system (in yellow in Figure 1), in which semiautonomous driving is possible thanks to the PC node.

In the proposed architecture, all nodes share the power system (in red in Figure 1), which consists of two lead–acid batteries (12 V each) and DC/DC converters. They are also interconnected and communicate through the CAN bus (in green in Figure 1).

The motor node (in blue in Figure 1) connects the power to the rest of the wheelchair, including the safety and regulation systems. In addition, the motor node is responsible for driving the DC engines that move the platform following a differential kinematics fashion. The mechanics, electrical, and electronics details about this node are further described in Section 4.2. Drive wheels are also equipped with optical encoders in both platforms, so odometric dead-reckoning is used as the basic relative positioning system in the navigation loop, as detailed in Section 5.2. Furthermore, using as a reference the speed commands from the joystick node and as feedback the odometric information, a speed linear control loop is also periodically performed in each motor node in a commercial microcontroller. Software details about this low-level control are further described in Section 5.1.

The joystick node (also in blue in Figure 1) tackles the basic human–machine interface (HMI) task, in order to command the platform and choose its operating mode (manual or semiautonomous driving). In the manual mode, the wheelchair speed and direction are controlled through different interfaces (analog, digital, and blow) adapted to the user’s diverse functionalities, as explained in the Section 4.3. In the semiautonomous one, this node is used just as a communication bridge with the PC node, as described in Section 4.5. Finally, the joystick node incorporates lights, switches, and keys in order to inform about the wheelchair state.

In the current configuration, the sensor node is included only in SARA, the adult’s platform. The sensors node is set up to capture and process information that may improve the reactive navigation when standing on odometric information, as explained in Section 4.4.

Finally, the high-level node may use a PC (any type), in the case of SARA, or it can be integrated on a single-board computer (SBC), i.e., a Raspberry Pi 4, in the case of KATE. On the high-level node, and on top of a GNU operative system, a portable Robotic Operating System (ROS) is installed, making the autonomous or semiautonomous driving possible. Specific kinematic models were designed for the two platforms presented in this work, and different portable navigation behaviors, which make use of standard autonomous robots’ navigation functions implemented in ROS, were implemented as detailed in Section 5.2. The user can select on a preloaded map the navigation goal, where the wheelchair will robustly and securely move using the information of odometry (encoders) and other sensors, when available.

The rest of the paper is organized as follows: Section 2 analyzes recent scientific contributions to the state of the art in this topic, in order to highlight the ones presented in this paper; Section 3 describes, in detail, the modular architecture drafted in the introduction, highlighting the adaptability and flexibility, which are the most important contributions to our proposal; Section 4 and Section 5 include a detailed description of the different hardware and software elements that make possible the architecture functionalities and characteristics specified that are demonstrated in Section 6; finally, Section 7 summarizes the conclusions and contributions of this work, as well as future work and research lines.

## 2. State of the Art

Recently, the advances in autonomous robotic systems have impacted society in the form of commercial autonomous cars. From another point of view, the existence of millions of people with reduced mobility skills prompted numerous research groups to apply those advanced techniques for the benefit of such potential users. This is achieved by trying to export the latest advances in robotics to the field of assistive technologies [8,9,10,11,12].

**Figure 2:**
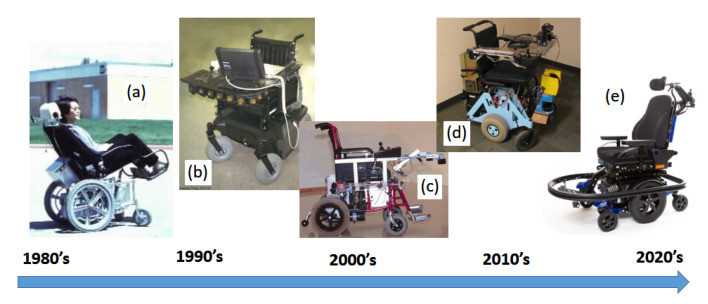
Intelligent wheelchairs timeline; some examples. (**a**) Head control [13]; (**b**) Navchair, with an ultrasonic ring for navigation [15]; (**c**) SIAMO, with voice commands and infrared safety sensors [1]; (**d**) Vulcan 1.0, from the University of Texas [17]; (**e**) Smart Rehab commercial wheelchair [18].

Already in the early 1980s, some works at Stanford University [13] began to apply ultrasonic sensors (USs) to an adapted wheelchair. Around the 1990s, other research teams around the world began their works in developing innovative mobility-assisting vehicles [14,15,16]. Those vehicles were similar in their initial conception to any standard mobile robot: a set of motors, wheels, sensors, and controllers. Those activities were spreading out throughout the world in the pursuit of the so-called intelligent wheelchairs (IWs). Some examples related to the IW timeline are shown in Figure 2.

Throughout these years, various surveys and reviews have summarized the evolution of research activities in this field. Among the most notable, we find the works of H. Yanco in 1998 [19], R. Simpson in 2005 [8], J. Leaman in 2017 [20], and Callejas in 2020 [21].

Recent studies [9,20,22,23] indicate that autonomous robotic platforms, as support systems for people with reduced mobility, would have a very positive impact on health systems and assistance to millions of people at national levels. However, after decades of work in research laboratories, only a reduced number of systems were able to become actual robotic chairs accessible to the group of potential users [3,24]. Few research-lab vehicles reached the market; from them, only a single-function model (line-tracking behavior) currently remains active from the remarkable SmileSmart Lab [18], but even with such small functionalities, its prototypes, specifically conceptualizedby therapists, are not affordable for users. It is thus concluded that we are still far from being able to transfer advances in mobile robotics to such groups, both for cost and safety reasons.

In his study [20], Leaman identified commercialization as one of the main problems in transferring these technologies to potential users. The need to adapt each solution to each case increases the cost of the product and decreases the business possibilities. On the other hand, the reliability and safety of these products is under discussion, since the tests are limited to controlled laboratory conditions rather than with real users in real environments [3,20]. Simpson, in 2011 [25], identified four problems as the cause of the difficulty in taking the intelligent wheelchairs (IWs) from the laboratories to the users:

- **Sensory system.** The type and cost of the required sensors must fit the nature of the application, mainly in order to detect and prevent dangerous situations.
- **Lack of a universal communication bus.** Most industrial wheelchairs currently use a proprietary intercommunication system. There is no open standard to guarantee interconnection and interoperability of modules and devices of different manufacturers.
- **Reimbursement.** It is hard enough to obtain reimbursement for a regular power wheelchair, and there is no evidence that intelligent wheelchairs improve outcomes. Therefore, manufacturers have no incentive to invest in this area.
- **Evidence.** There are not enough field tests to guarantee actual benefits from intelligent wheelchairs.

In our work, we intend to fill the gap between labs and users by proposing changes at the most basic levels of mobile platforms. As a result, we will focus on the first two factors of Simpson’s list, proposing an open bus architecture that will allow easier interconnections among different functional modules for very different user profiles, in such a way that production costs, installation, and maintenance can be reduced.

Moreover, within the research area of assisted mobility and robotic wheelchairs, incorporating autonomous navigation behaviors is a fundamental topic to be solved by the research community, as it is considered essential for applications in actual environments [10,11,26,27].

The most widespread solution adopted is the “Navigation-stack” [28], implemented on ROS [29]. This software package supports several variants, although the common architecture of all of them divides the navigation process into two parts: global and local planning. Global planning outlines a coarse strategy, useful when we are in large closed environments; in “Navigation-stack”, this function is performed by classical algorithms such as linear programming navigation (LPN) [30] or A* [31]. Local planning allows a fine strategy between points or intermediate goals marked by the global level, including the ability to navigate between objects not collected on a map. In the case of the “Navigation-stack” package, the algorithm used is the dynamic window approach [32].

Indoor navigation can use preloaded and/or self-generated maps (simultaneous localization and mapping—SLAM [33]), and relies on indoor positioning systems with active beacons (ultrawide band—UWB [34]) or passive ones (Apriltag [35]). Lately, the idea of enriching the information in the maps through semantic navigation (SN) has gained momentum, using artificial vision techniques. SN recognizes the environment and labels it with descriptive information [36,37,38], useful both for obtaining the position of the mobile device and having a human–machine interface (HMI) of higher level of abstraction. Moreover, and in order to increase the efficiency of autonomous navigation, deep learning techniques can be applied. In local navigation plans, they can be used to avoid moving objects by means of deep reinforcement learning (DRL) [39], using visual information provided by LiDAR (light detection and ranging) or a depth camera [40,41], or by fusion of sensory information [21,42,43].

However, in all cases, these techniques have a very high computational cost, unless edge computing architectures are used. Therefore, they are not really adequate for the objective of low cost that is pursued. In our proposal, this approach is addressed, always taking into account the cost objective, and therefore it is applied throughout the different levels of software layers and hardware modules that make up the developed robotic platforms.

The same problems were identified in [24], where the gap between labs and market was also highlighted, and our analysis in [3] is followed in order to obtain some first results of autonomous navigation within an ROS context, although in such work, usability loses importance compared to technical development, which is solved here with the modular architecture.

It has to be pointed out that despite the efforts in research laboratories, most of the designed prototypes require costly adaptations and fine-tuning procedures, both in software and hardware components, to be adapted to the specific environment of people with very reduced mobility. As a result, such autonomous behaviors do not have a broad commercial interest, and will never reach the market and the users.

Our work aimsto close this gap between manufacturers and users, proposing a modular and scalable architecture that, using the same basic software and hardware components, could be upgraded and adapted to any profile of users with reduced efforts and at a reasonable cost.

## 3. Proposed Architecture

The contribution presented in this paper is the creation of a modular IW architecture on top of a commercial powered one, and the validation of such a solution in two different platforms, able to be adapted easily to different profiles of users with different needs.

**Figure 3:**
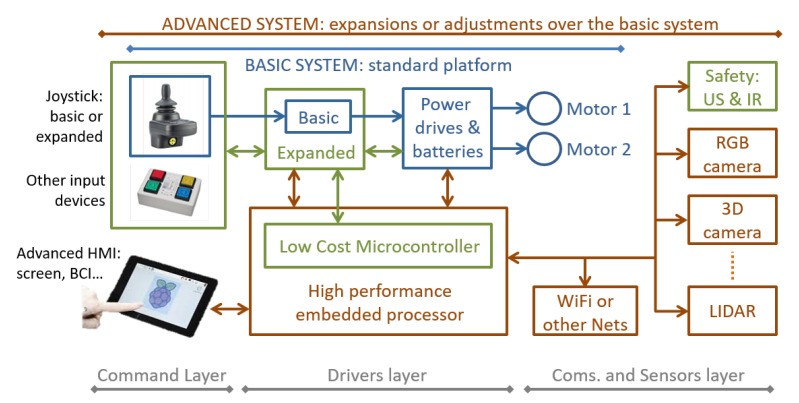
Block diagram of the intelligent wheelchairs (IWs) modular concept.

This concept is detailed in Figure 3, where it is possible to identify the different parts and elements of the modular solution. Several layers can be identified within the such concept, according to different criteria.

First of all, let us identify three levels of complexity and performance of the system, represented by the lines on top of the figure and with the colors of text and blocks:

- **Basic system**. Identified with blocks in blue and formed by the so-called standard platform. Apparently, it is just a standard electric wheelchair with just two motors, a power drive and a joystick, but such modules are fully programmable and already connected through a CAN bus.
- **Expanded system**. Its elements are identified in green. It is built on top of the basic system by upgrading or changing the joystick node with alternative input devices (i.e., switches, as shown). In addition, it can incorporate some safety or simple obstacle avoidance behaviors by adding range sensors (ultrasonic, infrared, or contact). This level of complexity can be considered just as an improved version of the basic system, but it is commercially interesting because of its added value: more performance at a very reduced cost.
- **Advanced system**. This level completes the architecture with the elements identified in brown color. Possible blocks include vision sensors (standard and 3D cameras), advanced range sensors (i.e., LiDAR), off-platform communications (WiFi and equivalents), and advanced HMIs. This level also requires more powerful processors and software.

Those levels of complexity are fully related to the objective of making an industrial production of IWs affordable, by means of using the same platform (a single main budget) to build up several different solutions, in terms of cost and performance, that are also able to be upgraded according to the evolution or needs of the same user. In the remainder of the paper, we focus on basic and advanced systems, taking into account that the expanded system, in terms of complexity, is just an improved version of the basic one.

From the point of view of the system builder, in Figure 3 there are also three layers of abstraction shown. They are identified with the lines at the bottom of the figure:

- **Command layer**. This is formed by the blocks on the left. This layer is composed of the HMI used by the person in the wheelchair to drive it. In the current prototype, while in the basic system, three different HMI behaviors were developed for manual operation of the platforms; those behaviors are detailed in Section 4.3. Other behaviors, mainly related to autonomous navigation, are included in the advanced system through the PC node, as detailed in Section 4.5.
- **Drivers layer**. This stands for the electronic control, computing, and power management modules, shown in the central area of the figure. Such a layer can be considered independent of the kind of system being implemented, from basic to advanced.
- **Communications and sensors layer**. This is shown on the right side of Figure 3. Sensing the environment is a crucial task for increasingsafety, improving navigation autonomy, or incorporating some kind of interaction with the environment itself. On the platforms presented here, we tested ultrasonic sensors, inertial measurement units (IMUs), LiDAR, and RGB and 3D cameras, as detailed in the following sections. Communicating with the environment (i.e., by WiFi) can be performed using standard modules connected with high-level processors when required.

Behind the overall structure of the system, shown in Figure 3, stands the physical communication resources among nodes and modules. High-level sensors connect to high-level processors using their standard interfaces (i.e., USB). This standardization is used, as well, for the off-system communications with the environment. Custom or basic nodes, such as joystick, advanced joystick, power drives, simple range, safety sensors, etc., communicate through a CAN bus. For SARA and KATE prototypes, we designed a set of messages (see Section 5.3), appropriate for controlling an autonomous wheelchair, that could derive a standard for IWs.

**Figure 4:**
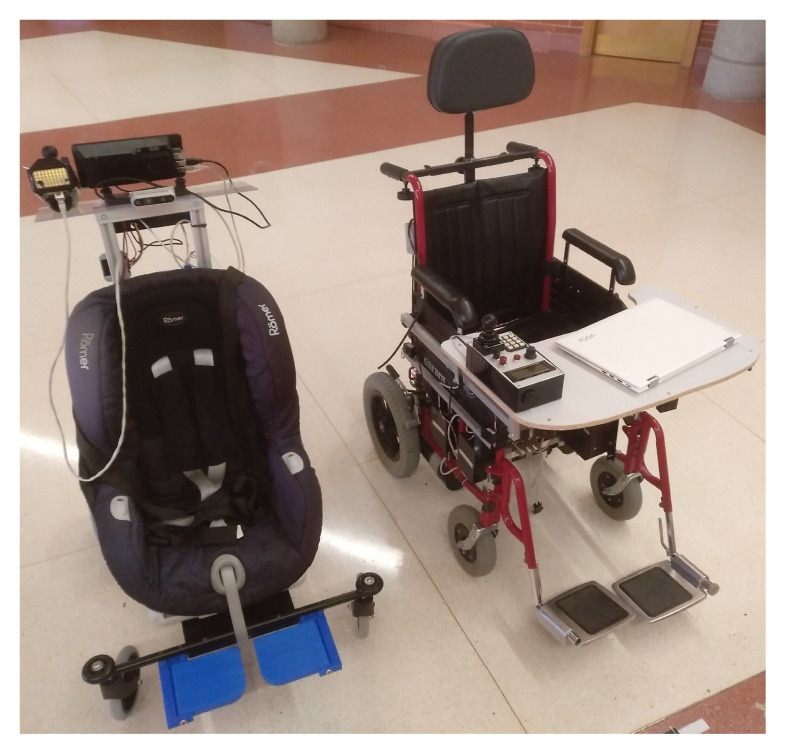
Picture of prototypes based on the proposed architecture: KATE (**left**) and SARA (**right**).

In Figure 4, both prototypes are shown: KATE, the one built up for children, is shown on the left; SARA, the one designed for adults, and the origin of this proposal [3], is shown on the right. Despite some differences between them (derived from the differences in power and control requirements), both systems were built using the structure described in this section.

As can be seen in the figure, the disposition of the HMI elements in SARA is designed based on the user itself, so they are located in front of him. However, in KATE, the HMI was placed mainly for its companion (relative or caregiver); this configuration was adopted for testing purposes, but it is not a must.

## 4. Hardware Modules and Nodes

In this section, we describe the hardware components that form the actual IW system implemented and tested on SARA and KATE prototypes. The complete IW architecture proposal was described in Section 3, and Figure 3. However, in this paper, we focus on the actual structure of nodes and modules developed and tested in SARA and KATE, which forms a subset of the complete concept and can be simplified to the reduced block diagram shown in Figure 1.

The following subsections detail the main characteristics of the developed nodes, as well as their interconnection through a common link for data and power, called intelligent wheelchair bus (IWBus). Altogether with those details, we show and compare some changes between platforms derived from their specific applications and desired performances and behaviors. A set of tests and results, achieved during the development of the nodes, are also shown.

### 4.1. Intelligent Wheelchair Bus (IWBus)

A key element of any distributed system, such as the one proposed here for an intelligent wheelchair, is the definition and implementation of its interconnection media, both at a physical and functional level.

Most manufacturers use their own communication bus, such as the Control Dynamics DX Bus [44]. Some projects in IW used this interface [45,46,47] to send driving commands to the wheelchair. However, such an interface has several problems, most of them stemming from the closed nature and limitations of its specifications. One of the most important problems is the presence of high system delays that affect dynamic performance, such as those required by obstacle avoidance tasks. To solve this problem, Canoz et al. in [24] proposed a high-speed microprocessor connected to various safety sensors via a high-speed CAN bus. Other researchers also use secondary buses (RS485) to exchange information between the different functional modules [47].

However, in all cases, the wheelchair behaves similar to a “black box” (receiving only commands), operating in open-loop mode, which reduces the potential benefits of using high-level control algorithms. In addition, the use of the wheelchair proprietary interface is highly dependent on company policies and can be greatly affected by their changes. As an example, control dynamics has ended the life of DX-related products and is not recommended for use in new designs [48].

**Figure 5:**
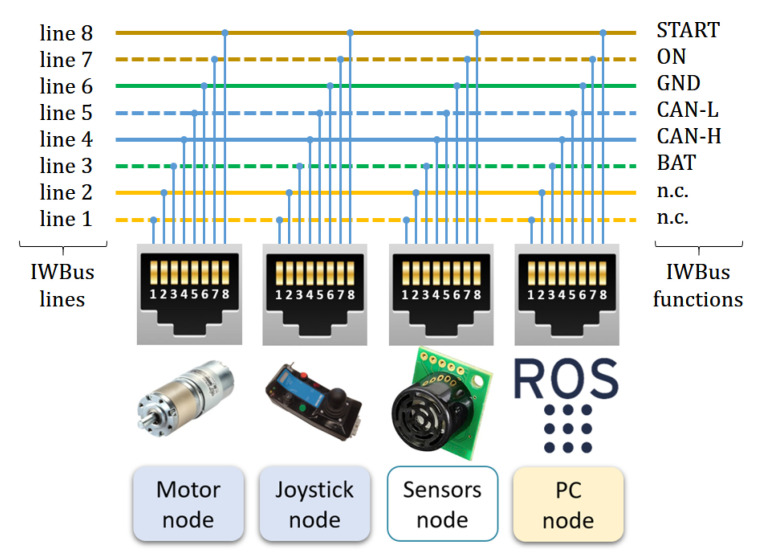
Detail of the CAN bus lines used to interconnect the nodes.

Those facts make clear the benefits of having an open standard for accessing all power wheelchair resources and being able to receive commands and send status information. The definition of such a standard is one of the objectives of our work: an architecture that wheelchair manufacturers can accept and that can be defined and maintained by the entire developer community. As a result, we defined, using standard components, the intelligent wheelchair bus (IWBus) shown in Figure 5. This figure also shows the hardware modules and nodes that will be detailed in following subsections.

The purpose of the IWBus is to transfer power and information among system nodes. Quick connection among nodes is made using RJ45 connectors and Ethernet cables. Those standard connectors and cables are suitable for the required functions due to their versatility, low price, and maximum current transmission specifications.

There are eight lines available that were assigned to the desired power distribution and CAN communication functions as shown in Figure 5. Wire colors identify their pairing according to Ethernet standards. Though such pairing is not mandatory for low-speed signals and power distribution, this is essential when connecting CAN bus data lines (CAN-H and CAN-L), which are connected to the pair of lines 4 and 5. The required termination resistors in CAN lines are not shown, because their location depends on the actual position of the initial and final nodes connected to the IWBus; those resistors can be connected, or not, using jumpers. At the physical level, standard CAN drivers are used.

The DC power distribution is made through paired lines 3, labeled BAT (carrying 24 V) and line 6 as GROUND, 0 V. Using type 3 cables in IWBus will allow a maximum current per pair of 600 mA. As a consequence, the overall power consumption of all the nodes that could be fed up through IWBus is about 14 W. This amount of power is enough for current low-power processors and devices.

Two more signals in IWBus are required to manage power initialization and distribution among the electronic modules connected to it: signal START, in line 8, and signal ON, in line 7. The START signal comes from a closed contact in the “Power-On” main switch; this contact will drive a power relay that closes battery contacts for powering the overall system. Finally, the ON signal works similar to another switch-to-ground output that, in this case, is used to confirm to the rest of the system nodes that power initialization was correct.

An important exception in power distribution through IWBus are those nodes that require controlled shutdown procedures or have relatively high power demands. This is true for PC nodes or similar ones. In such cases, IWBus is only used for communication and vehicle state monitoring. In SARA, the PC node has an additional DC/DC converter, directly connected to the battery, so the ON signal on IWBus can be used to know if the rest of the nodes connected on IWBus are also powered.

IWBus on SARA and KATE used six out of eight RJ45 lines. Lines 1 and 2 were left unconnected (n.c.) in order to prevent heavy failures when cabling. In future industrial versions, where those kinds of problems are minimized, better arrangements of cables and connectors will be defined.

### 4.2. Motor Node

The minimum IW system requires the following components: two processor modules (motor and joystick nodes); an interconnection subsystem, the IWBUs; a power battery; and two motors for implementing a differential drive platform. In this subsection, we will focus on the motor node.

**Figure 6:**
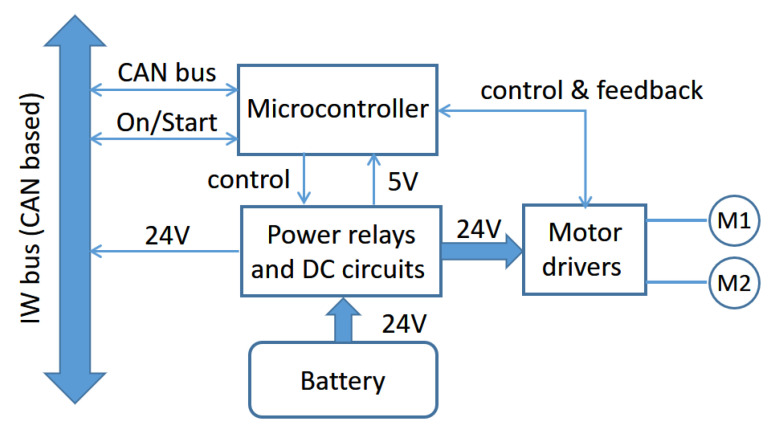
Elements and functions of the motor node. Connections to signals in IWBus are also shown.

The two main functions of the motor node are managing power distribution, from the battery to the rest of the nodes in the IW, and controlling the DC motors. Figure 6 shows a block diagram that illustrates the different elements, components, and functions developed by this node.

The power comes from a DC battery of 24 V made up of two 12 V batteries of 26 A/h, in the case of SARA, and 7.2 A/h for KATE. The processing core is based on a low-cost LPC2129 CAN QuickStart microcontroller board, which carries on the following set of tasks:

- Receiving and analyzing the corresponding encoder pulses from both DC motors, M1 and M2.
- Driving the motors, using a bipolar PWM strategy.
- Computing a PI speed control for each of the motors.
- Exchanging and processing status and command messages coming from CAN lines in IWBus.
- Managing distribution of battery power to motor drivers and the rest of the system module, using the IWBus.

From the system point of view, the motor nodes of SARA and KATE prototypes all behave in the same way: the same commands, the same power distribution, and the same dynamic functions. However, from the inside, both nodes have important differences due to the differences in size, weight, and desired cost between their specific applications (adults or kids assistance).

One difference is regarding the motor drivers. The adult’s version, SARA, has a Roboteq Power Board AX3500 with serial communication and an integrated PID controller. The kid’s version, KATE, has lower power requirements and, in this case, it is equipped with cheaper LMD18200 drivers, excited with the corresponding PWM signals generated by the microcontroller itself, as a result of a PI speed controller.

Another difference is the encoder resolution. SARA had installed, on each motor axis, an HEDS-5500 encoder of 500 pulses per revolution and two channels, combined with a 1 x 32 reduction gearbox. Having such a huge angular speed measure resolution, control algorithms can perform accurately. However, KATE, conceived as a low-cost platform, had installed low-resolution encoders that present practical problems for the speed control algorithms. The encoders attached to KATE motors have a resolution of just seven pulses per revolution, within two different channels, so just 28 pulses by revolution are available for completing the angular speed control. Section 6, shows details of the difference between both (SARA and KATE) encoder resolutions.

A low resolution from motor encoders limits the information sent to the controller to implement the required platform odometry. This issue is an intrinsic and standard problem found when working with low-cost robotic platforms. Odometry problems cause position and speed errors, reverting to a bad localization in the platform navigation task. As a partial solution, this problem can be addressed by increasing the sampling time of the velocity controller, a technique that is described in Section 5.2.

In our current work, the noisy encoder information available in KATE is compensated using a special reactive methodology, using laser scans, in a probabilistic framework for localization (AMCL [49] algorithm), as described in Section 4.5.

### 4.3. Joystick Node

**Figure 7:**
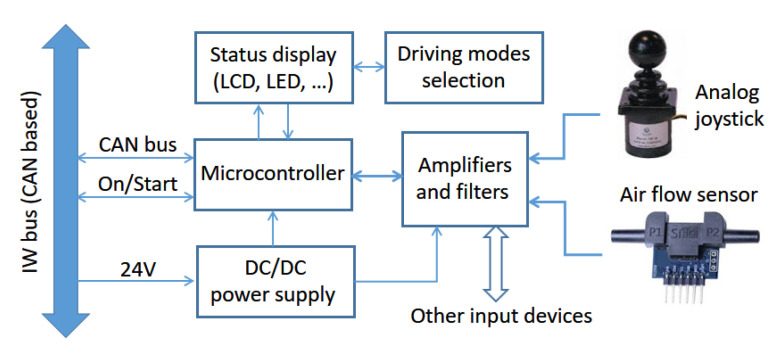
Elements and functions of the joystick node and its interconnection to IWBus.

The joystick node is the second key component of a basic system and, together with a single motor node, allows the construction of a platform that, having identical performance to a conventional powered wheelchair, will also have all the potential benefits of being easily upgraded to advanced behaviors (Section 3). Figure 7 shows a block diagram of the elements, components, and functions included in this node.

The joystick node was programmed to enable users to manually operate the platform in several easy-to-drive modes, with little, or even no, need for other nodes. Its main functions are the following:

- Platform direction control.
- Platform maximum speed selection.
- Switching on and off the IW.
- Battery power level display.
- Operating mode selection and display.

#### 4.3.1. Joystick Building Blocks

The processor is the same low-cost board based on an LPC2129 microcontroller, so the core code can be the same as the one in the motor node, reducing development time and cost. A DC/DC converter transforms 24 V into the lower voltages required by joystick node circuitry with low power losses and improved efficiency. As input devices, the ones tested in SARA and KATE were an analog and linear joystick and an airflow sensor, whose functionalities will be described later. Some analog amplifiers and filters are also required for adapting sensor signals to the specifications of analog to digital converters of the microcontroller. Finally, the user can know and set different behaviors or modes using buttons, switches, or a small keyboard as inputs, and an LCD display or some LEDs as outputs.

**Figure 8:**
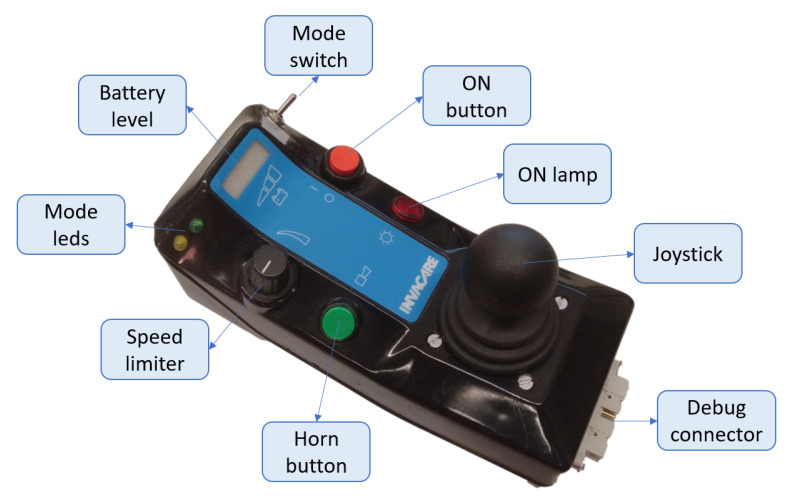
IW joystick node: components, status displays, and controls.

Figure 8 shows an external view of the joystick node in KATE. We used the case of an old commercial powered wheelchair, just for practical reasons: most users identify the common functions of other joysticks and may drive the chair easily after a few minutes of training. For instance, the speed limiter, shown in the lower left side of the joystick module (Figure 8), allows users to set the maximum speed manually, so they obtain the required driving skills for any new driving mode with improved confidence. The available driving mode is set by simple switches, and dedicated LEDs confirm the selected mode.

The SARA joystick module (not shown in this paper) has almost identical functions but with a bigger case that was adapted for being mounted on top of a small table in front of the user. In this case, a small keyboard is used to select driving modes and other configuration parameters, while an LCD display is used to exchange information between the system and the user.

#### 4.3.2. Direct Driving Modes

Direct driving modes are those where the user can drive the wheelchair by themselves, with no need for additional machine support. That function is developed by standard powered wheelchairs, but such simple behaviors must be included in the concept behind IWs and their specifications in order to make wheelchairs builders interested in such products and businesses [3].

As a demonstration of our design concept, we implemented up to three different operation modes just for the basic system, the one with only two nodes. These modes are analog joystick, digital joystick, and blowing joystick.

1. **Analog joystick**. In this mode, the wheelchair movement is controlled directly with the joystick position, just as in a conventional powered wheelchair.
2. **Digital joystick**. A set of specific actions over the joystick are translated into commands of linear and angular speed using an internal state machine. This mode was designed for people having problems making stable and continuous moves on a conventional joystick.
3. **Blowing joystick**. The input device in this mode is the linear air flow sensor. A user with severe hand or arm mobility problems can drive the chair easily by blowing through such sensor, using a flexible small tube. In a similar way to the **Digital joystick**, specific blowing actions are translated into commands of linear and angular speed using an internal state machine.

Digital and blowing joystick modes were developed according to a single lemma: let the user directly drive the wheelchair while it is possible. This lemma relays on the fact that the best and the most intelligent processor on top of any kind of wheelchair is the human being. Therefore, direct or moderated assisted driving are the preferred controlling modes over automated ones.

Digital joystick mode is appropriate when it is difficult for the user to maintain a continuous force, pulling or pushing the joystick during forward (or reverse) displacements. This is a convenient mode when the arm and hand muscles of the user have reduced capacities or cannot develop constant forces. The digital joystick was programmed as follows:

- Beginning with a stopped vehicle, moving the joystick **forward** will increase linear speed **forward**. Returning the joystick to the center position, or stoppingpushing, will maintain the acquired speed.
- Beginning with a stopped vehicle, moving the joystick **backwards** will increase linear speed **backwards**. Returning the joystick to the center position, or stopping pushing, will maintain the acquired speed.
- When moving forward or backwards, a momentary action over the joystick will increase or decrease speed, depending on the direction of such action.
- When moving, if linear speed reaches zero, the wheelchair will remain stopped until a new action confirms the desire to change the forward/backwards behavior.
- When moving, an intense and quick change in joystick input will stop the chair (used as an emergency stop).
- When moving, no action over the joystick will maintain linear speed. This behavior allows the arm and hand muscles to partially recover from dynamic efforts.
- To correct the driving line turning right or left, actions must be made to each side. Angular speed is constant and is set to zero when the corresponding action is over.

The blowing joystick behaves similarly, following the design guidelines of being easy to manage by most users. Blowing and digital joysticks have been tested by many different users, while the SARA prototype has been shown in different demonstrations and exhibitions. All of them needed just a few minutes of training before being able to drive the wheelchair by themselves, even at high speeds (roughly up to 2 m/s). A short video of one of such exhibitions can be seen in [50], which was shot at the UAH Open Day 2019 show.

### 4.4. Sensor Node

The sensor node is responsible for managing low-speed, low-resolution, or simple safety sensors, able to be controlled by a low-cost microcontroller. Such kinds of sensors are ultrasonic or infrared range detectors, mostly used to avoid surrounding obstacles, and inertial measurement units (IMUs; many of them available as single chips solutions) used to reinforce positioning algorithms and able to detect collisions as well.

**Figure 9:**
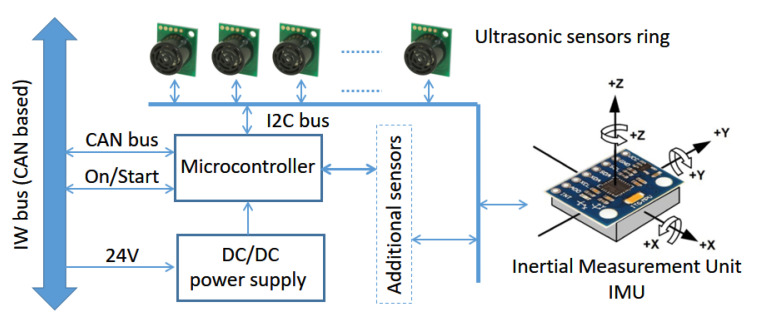
Block diagram of the sensor node, showing the ultrasonic ring and IMU.

Figure 9 shows a block diagram of the current sensor node available in SARA. As in other basic nodes, the processing core is an LPC2129 microcontroller board, connected to the overall system through the IWBus. In its current implementation, this node was equipped with an ultrasonic ring of nine RF02 sensors and one IMU, MPU-6050, measuring six variables (acceleration and gyro rate on each of three 3D axes). The controller and all the current sensors are connected to a single I2C channel.

At the system level, this node has to manage and synchronize the flow of data coming from the installed sensors. This is achieved in two directions: on-board, assigning time-slots and sampling sequences to each group of sensors; off-board, filtering and preprocessing the flow of data prior to sending it through IWBus.

As KATE is usually driven by an adult caregiver, the sensor node is currently assigned to SARA. Ultrasonic and IMU information is used in SARA in two different environments:

- Without a PC node, where the main controller in the system is the joystick node, such information is used to implement simple behaviors, such as obstacle avoidance and collision detection. This is useful when assisting the user in special drive modes, such as digital or blowing sensors.
- With a PC node (see Section 4.5), ultrasonic ring and IMU information can be included in navigation algorithms in order to improve their performance.

**Figure 10:**
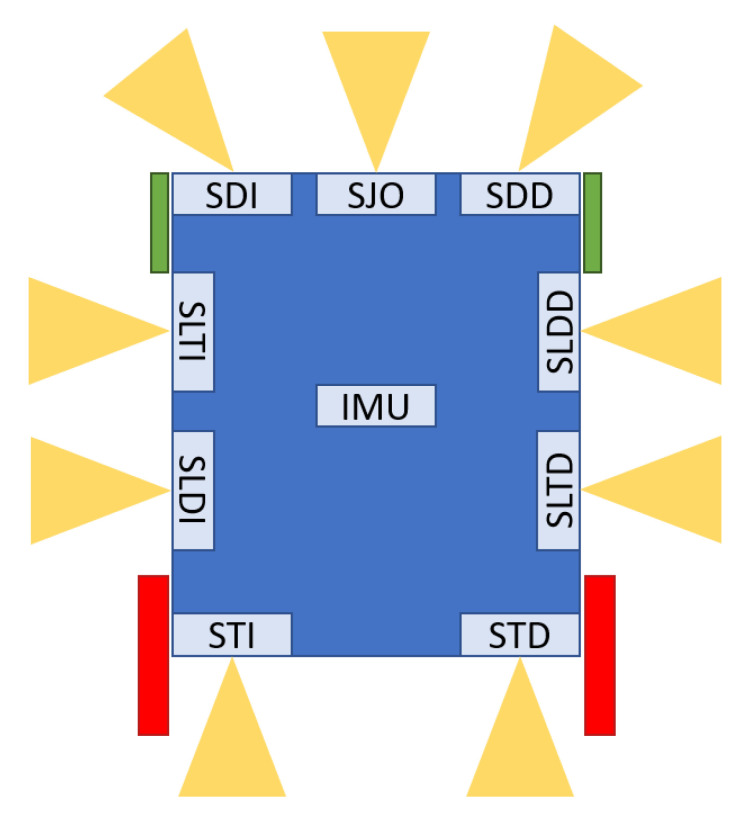
Sensor distribution on SARA.

Figure 10 presents a schematic diagram of the distribution of the sensors around the SARA vehicle. The nine ultrasonic sensors were distributed around the vehicle perimeter; their names are acronyms of their location (in Spanish). In trying to minimize range errors coming from echoes among sensors, while keeping a reasonable US system sampling time, only two cycles of 100 ms period are used [2]. One cycle begins with SDI, SDD, SLTD, STI, and SLTI sensors; the next cycle completes the exploration by triggering SJO, SLDI, SLTD, and STD sensors. Because of their relative position and taking into account the coverage area of each SRF02 sensor, the interference is not important for IW functions.

Sensors information is packed and sent through the IWBus, according to the protocol and the CAN messages defined for the system (see Section 5.3).

### 4.5. PC Node

**Figure 11:**
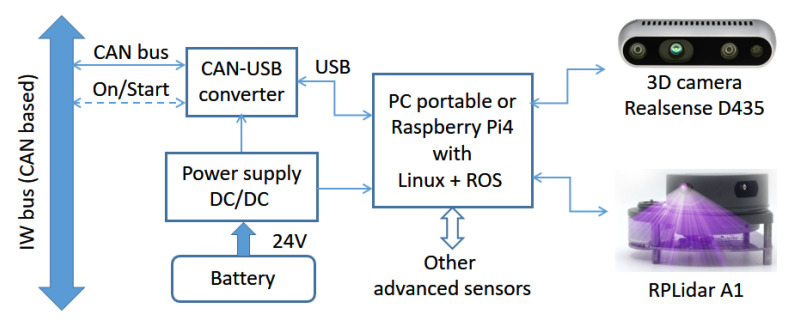
Block diagram of the sensor node, showing the ultrasonic ring and IMU.

This node is responsible for autonomous and semiautonomous navigation. Because of that, it is only required when such kinds of behaviors are needed. This is the case when users have heavy mobility problems and cannot drive the wheelchair with an appropriate level of safety and autonomy. Figure 11 shows a block diagram of the PC node tested on SARA and KATE.

The core of the PC node is the processor. In KATE, this is a single-board computer (SBC) with a Raspberry Pi 4, a device leader in power, dimensions, and cost within this type of commercial high-level processor board. In SARA, the processor is a portable PC. The standard human–machine interface (HMI) for both platforms is a touchscreen.

Additionally, in both solutions (SBC and PC), the operative system selected to develop and bear the autonomous navigation is ROS, installed over a Linux Ubuntu 18.0 distribution. Nevertheless, although the interface and control algorithms are basically the same, there are important implementation differences in the installation of software systems and packages; however, the specific procedures and implementation issues fall out of the scope of this document.

When implementing navigation behaviors, a set of suitable high-performance sensors are needed; this is in order to make the detection of obstacles and structures of the surrounding environment of the platform possible. The sensors used in this work, and shown in Figure 11, are a 3D camera, Intel Realsense D435 model, which offers RGB and depth information, and a laser rangefinder, LiDAR, model Slamtec RPLidar A1. Both sensors were chosen to take into account the constraint of using low-cost technologies, to maintain a reasonable system cost of a possible commercial solution. The chosen devices make it possible to deliver a very reliable recognition of the environment while keeping, at the same time, affordable prices.

PC-like processors (both a portable computer and a Raspberry Pi) require controlled shutdown procedures to prevent operating system corruption. As already mentioned, these restrictions force the wheelchairto have a separated power supply system (as seen in Figure 11). Communications with the wheelchair platform must be performed through its CAN lines, via IWBus. Because the standard serial communication ports in PC-like processors are usually USB ones, we also added a protocol converter that transforms the CAN messages into serial ones, suitable to be connected to any USB port.

## 5. Software Architecture

This section provides a logical description of the system, explaining how the software characteristics are implemented and how they work. Examples and results that were achieved during the development of the nodes are also presented in this section.

Software layers and resources are directly related to the level of system complexity, which also depends on the set of behaviors implemented in the actual system. We offer different modes of usability in our platforms, but, basically, they can be classified into two main groups:

- **Direct driving**. In this mode, the IW can be driven directly by the user, without requiring PC nodes and complex algorithms and sensors;
- **Computer-assisted driving**. Those modes are only required when the users have strong difficulties controlling and/or managing the IW, so the added intelligence of a PC controller is needed.

In direct driving modes, the user just needs a basic system that consists of motor and joystick nodes. Keeping full control of the vehicle, the user can also shift to an advanced system when incorporating a sensor node, for safety or assisted driving purposes. Those modes were already presented when describing joystick node functionality (Section 4.3).

In the following sections, we focus just on the autonomous or semiautonomous modes, which require resources only available through PC nodes. Nevertheless, the first subsection presents the low-level control algorithms that run on the motor node controller. The next subsection details the autonomous navigation resources. The current section finishes by describing the main aspects of the communications parameters.

### 5.1. Speed Control

**Figure 12:**
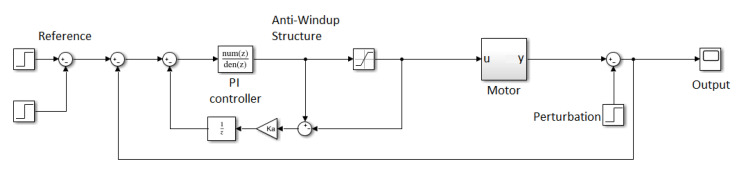
Block diagram of the DC motor angular speed controller.

The processor in the motor node is responsible for driving the platform DC motors. It runs a proportional integral (PI) software controller that is designed to nullifyangular speed error in steady state. The block diagram of the controller is shown in Figure 12.

The motor block corresponds to the partof the system that must be defined. We modeled it using Equation (1) and then used the zero-order hold method to discretize it.

(1) $$G\left(s\right)=\frac{A}{\tau \;\cdot \;s+1}$$

*A* represents the speed constant of the motor, with an estimated value of (187.5 rad·s^−1^·V^−1^), and τ is the time constant measured experimentally (approximately 65 ms). Those are the values corresponding to the SARA platform.

The control of the system is modeled with the standard transfer function of a discrete PI controller, designed in MATLAB. The PI controller is represented with Equation (2), where *K* = 0.132, *c* = 0.579, and *p* = 1.

(2) $$H\left(z\right)=K\;\cdot \;\frac{z-c}{z-p}$$

In this structure, we add an anti-windup controller to reduce the saturation effects. The value of the anti-windup controller constant Ka is 10. After the design stage, it is necessary to write it down into code. Such code is downloaded onto the microcontroller and it is applied within a cycling scheduler with a sampling period of 10 ms.

**Figure 13:**
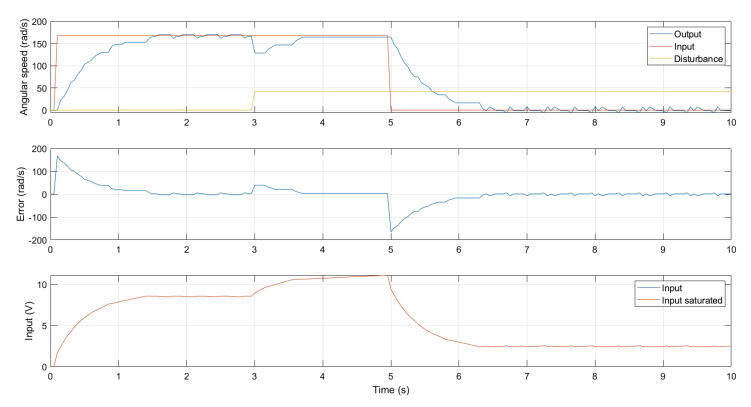
PI low level speed controller behavior.

This simple controller belongs to the lower level of a standard mobile robot platform. Its function is only to nullifythe speed error in the steady state. Figure 13 shows details of the PI controller performance after an actual run. Note that, over this control layer, a higher level of abstraction allows the implementation of the controllers implemented by ROS. This high-level layer is explained in Section 5.2. In this context, it should be noted that nonlinear approaches, such as MPC controllers [51], could improve the current behavior of the speed control of wheelchair DC motors. Unfortunately, as mentioned, any direct handling control is deeply occluded by the higher-level path control included in the ROS “Navigation-stack”, allowing us to obtain better results with such a simple controller.

Both platforms, SARA and KATE, share this low-level layer for controlling motor speed, though only the figures for SARA were given here.

### 5.2. Autonomous Navigation Resources

To implement autonomous navigation in our IW, we chose ROS because it supplies utility libraries, called “packages”, that can be easily added to every robotic project. Thus, it is possible to install the packages needed for the platform; the most important are the following:

- CAN communication [52].
- Navigation, localization, and mapping [49].
- Speed and acceleration controller [53].
- Camera information [54].
- LiDAR information [55].

Within each controller loop, the PC first communicates with the platform, via IWBus, in order to receive information about the encoders’ readings: the vehicle’s current linear and angular speed. With this information, and using laser measures coming from the LiDAR sensor, the platform is located on the environment map, which must be previously loaded. Then, using depth information coming from a 3D camera, it is possible to detect obstacles and avoid them, making corrections to planned trajectories. At last, once the path is calculated, the respective speed values of the wheels are sent back to the motor node via IWBUs.

**Figure 14:**
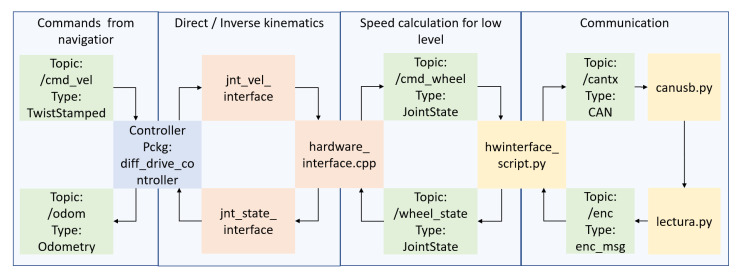
General scheme of high-level control.

This high-level process is summarized in Figure 14, showing the scripts, packages, and topics used and their relationships. Block colors represent the type of elements. In green, we have ROS topics, which are internal messages of ROS; in blue, the ROS packages; orange blocks represent methods and C++ scripts; finally, in yellow, we identify Python scripts.

In the rightmost part of the diagram, we represent the external communication processes with the low-level layer (motor node). We send the information of the topic cantx through the CAN bus and we receive the CAN messages from the platform using the canusb.py script. The lectura.py script translates the CAN messages to the topic and publishes them.

Between the hardware_interface files, we use the previous information to calculate the speed of the platform and the commands for every wheel. The jnt_interfaces methods apply the different robotic kinematics to set a point of understanding between the navigator and the platform, stabilizing the position and speed of every joint of the robot.

Finally, the navigator receives information of the odometry and publishes the cmd_vel topic, which are the linear and angular speed commands for the robot.

**Figure 15:**
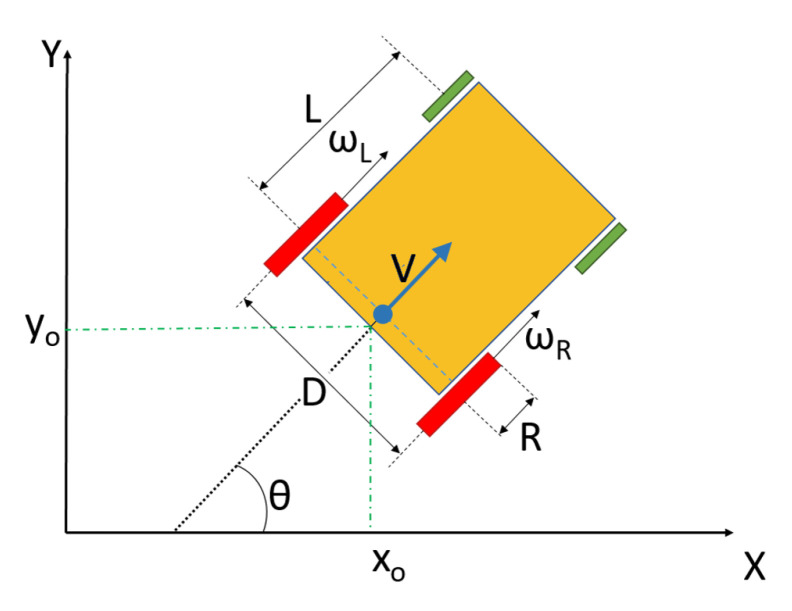
Cinematic model of a wheelchair.

On these platforms, the following consideration is very important, because these wheelchairs do not have the typical structure of a differential robot. They have two free wheels at the front that cause odometry noise and some changes in the cinematic model, as shown in Figure 15.

Equations (3) and (4) describe the direct kinematics used with odometry in the navigation process, in order to obtain the wheelchair’s position over time through a dead-reckoning standard process, described in Figure 15.

(3) $$\omega_{D}=\frac{1}{R}\;\cdot \;\left(V+\Omega \;\cdot \;\frac{D}{2}\right)$$

(4) $$\omega_{I}=\frac{1}{R}\;\cdot \;\left(V-\Omega \;\cdot \;\frac{D}{2}\right)$$

Here, Ω is the angular speed to be integrated to obtain the wheelchair angular position θ.

### 5.3. Communication Parameters

The communication of commands and status parameters of the IW is achieved through the CAN [56] lines within the IWBus. Using CAN resources fulfills the main objective of reducing the costs of the system upgrading by using standards, instead of the higher costs of custom solutions.

Each message in CAN will carry a payload of 8 bytes of data (excluded protocol overhead), divided into two blocks of 4 bits (32 bits length), called message part A and message part B. The information sent over the IWBus is divided into specific fields, standardized to 8 or 16 bits for each field, depending on the type and resolution of the data being sent. The data payload is packed with protocol overhead bits that include a message identifier, ID, data size information, and CRC field, for error checking. The ID field of CAN messages is filtered by the microcontroller hardware, so each node only reacts and reads the messages whose message ID is significant to it.

Table 1 shows details of the structure and fields of each of the messages that are sent through current implementation of IWBus. Cell size represents the data length of each field, the minimum being 1 byte; data fields have fixed lengths of 1, 2, and 4 bytes, as shown. The range information, coming from the US sensors, are identified following the naming convention shown in Figure 10.

The flow of messages over IWBus is as follows. When the platform is switched on, the first node used in transmitting a message is the joystick node (ID 0x110), sending the X–Y joystick values, angular speed set-points, calculated from those joystick values and the working mode of the platform. Such information is sent every 100 ms, which is the basic platform sampling time.

The motor node starts transmitting messages (IDs 0x101 and 0x102) at sampling time; in order to guarantee the appropriate extraction of actual wheel speeds, an absolute timestamp (in ms) is sent with every message. In SARA, the voltage level of the battery is also sent (ID 0x210). When installed, the sensor node sets the IWBus ultrasound and accelerometer measures (IDs 0x201, 0x202, and 0x203), when available (Section 4.4).

If the platform is in PC mode, the angular velocities generated by the joystick are ignored and the PC node transmits its own commands (ID 0x120). System messages are published periodically, at different rates that depend on the origin and/or the destination of such information. Messages rates are shown in Table 2. The message with ID 0x111 is not present in this table, because it is not a periodic one: it is sent just when the main control mode is changed from joystick to PC, and the reverse situation.

In order to check the uniformity of messages generation, the IWBus was monitored over time to have enough measures. In Table 2, the column **counts** reflects the number of received messages of each type to obtain the variance of the sampling rate, which is shown in **variance**. The last column is **the percentage of occupation** of the IWBus; it was computed after the CAN bus data rate, of 1 Mbit/s, and the number of bits of each message.

The message published by the PC node (ID 0 x 120) is the one having the highest variance, compared with the others. Such a difference comes from the different processor resources and computing load: while microcontrollers create and publish messages using interruptions and internal timers, the PC depends on ROS, with its own internal scheduling, to publish them. This is an important factor to be taken into account, as itprocess noises, when designing high-level controllers.

## 6. Results, Vehicle Performances, and Functionality Analysis

In this section, we present the functional results obtained with the developed platforms. Two main aspects were evaluated up to here: the capacity of the serial bus included in IWBus to give service to IW modules; and the performance of the wheelchair prototypes taken as standard mobile robots. After those results, we will check the suitability of our proposal to be a platform able to cover the broad range of needs of people with disabilities: from the most basic and general ones to the most specific and advanced system.

### 6.1. IWBus: Communication Results

When working with a distributed system, where its functions rely on the exchange of control and status information through a serial data link, bus occupancy and transmission delays are key factors that affect overall system reliability. In the current section, we show, with experimental data, that IWBus have enough capacity for the specified IW functionality.

In Section 5.3, we showed the structure of a single message, which is capable of carrying 64 bits of data coding the required information to manage the IW. As the CAN bus speed was configured to 1 Mbit/s, taking into account the periodicity of each message, the occupancy of IWBus is very low. With the data shown in Table 2, we may see that the biggest figure is less than 0.12%, which makes the evaluation of possible bus overload worthless.

**Figure 16:**
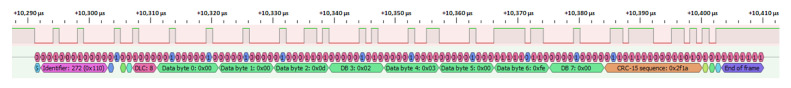
Detailed view of CAN message with ID 0x110.

Figure 16 shows a capture of a single full CAN message, with ID 0x110 (joystick status). Message duration is about 120 μs.

**Figure 17:**
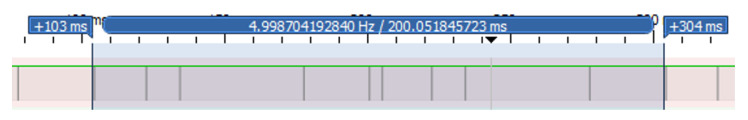
CAN bus occupancy in a full US ring processing cycle of 200 ms.

We also checked the time that it takes to receive a complete sequence of all CAN messages in the system proposal. This measure is presented in Figure 17 and corresponds to the duration of the longest period, 200 ms (ignoring the period of the message of the battery voltage, which takes 2 s).

Figure 17 shows the IWBus traffic along a period slightly bigger than 200 ms. The CAN line shown here is idle when high, so the low spikesindicate the occurrence of a CAN message. The sequence of messages here shown corresponds to the ones found between two consecutive US measurements of the same group; as stated in Section 4.4, USs are fired into two groups each 100 ms, so each group sends its information every 200 ms, which is the time lap remarked on the figure. Every other IWBus message appears between initial and final marks (except battery voltage, which reports every 2 s). We confirm the extremely low occupancy of the CAN bus resource and its availability for further tasks.

### 6.2. Platform Navigation Results

In this subsection, we show some results of the navigation skills obtained in SARA and KATE by using standard ROS tools. Many research centers and teams have contributed, based on ROS, to building and improving many different tools and utilities for robotic platforms and, specifically, for mobile robotic ones.

However, due to the evident heavy mobility limitations of potential users of high-level navigation strategies, the appropriate design of advanced HMI is the real keystone in the assisted mobility research field. Nevertheless, no matter how advanced an HMI is, it cannot be used if there is no commercial vehicle available for it.

As a consequence, the navigation results and measurements included here were intended as an actual demonstration of the behavior of the mobile platforms and their connectivity to the ROS components. If such a connection is correctly implemented, any ROS improvement can be directly ported to the proposed IW architecture, described in Section 3.

#### 6.2.1. Local Planner Results

**Figure 18:**
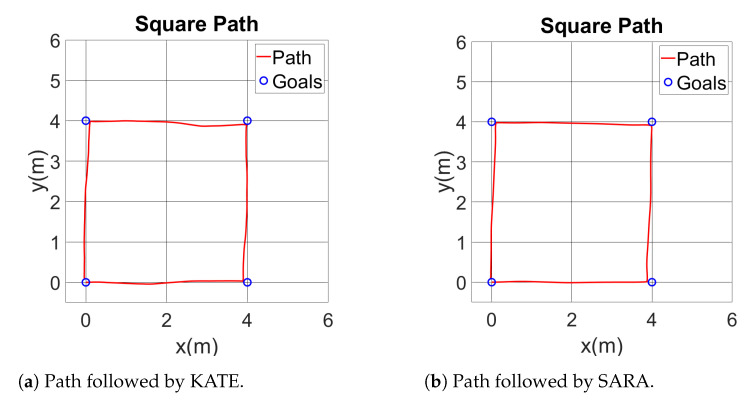
Comparison of square paths followed by SARA and KATE.

To study the local navigation skills, we set a square path (4 × 4 meters) to check different characteristics of the movement of the wheelchair. It is possible to see the dead-reckoning path followed by both proposed platforms in a square commanded one in Figure 18, obtained from the odometry readings.

The desired path begins on origin (X=0,Y=0) and with the wheelchair pointing up, that is, 90, and follows the square shape clockwise. Therefore, the sequence of points is (0,0), (0,4), (4,4), (0,4), and it finishes again on (0,0).

**Figure 19:**
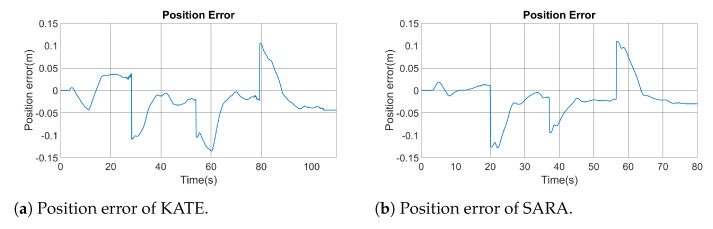
Position errors following the square paths.

Figure 19 shows the instantaneous positioning error from the square experiment on both platforms. Such an error is displayed as the deviation measured left (negative) or right (positive) from the commanded path. Note the discontinuities on error graphics due to turning points. As seen in both figures, the commanded and executed paths are very similar, both in KATE and SARA, with maximum deviations close to, but lower than, 15 cm.

**Figure 20:**
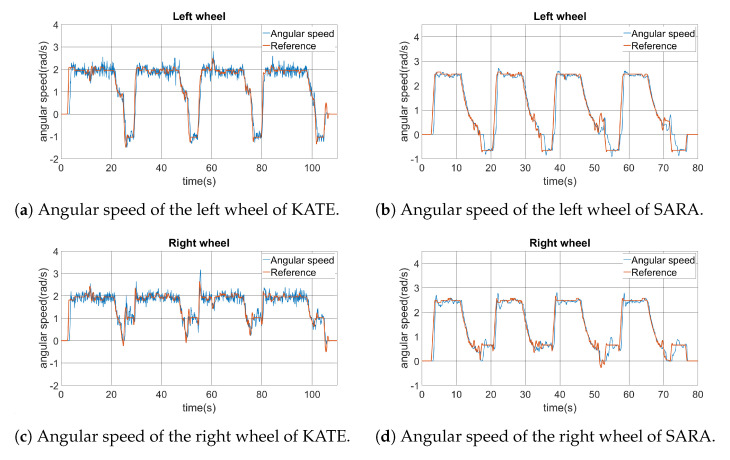
Comparison of angular speed between KATE and SARA left and right drive motors.

For the square trajectory, the angular speed of both wheels was also recorded and they are shown in Figure 20. Here, differences between KATE and SARA become evident. It is possible to notice a bigger quantification error noise on KATE’s positioning control. Nevertheless, both platforms show appropriate concordances between actual angular speed and the commanded reference on both wheels.

From the vehicle behavior, and after the analyzed data, it can be concluded that the low-level PI speed controller (see Section 5.1) works properly and it performs as planned.

**Figure 21:**
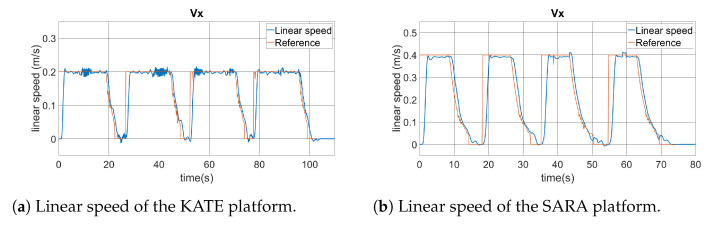

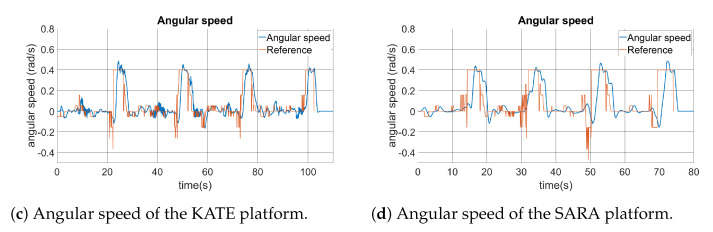
Comparison of the KATE and SARA platforms’ velocities.

Finally, Figure 21 shows the linear and angular velocities of both platforms while performing the square path test. This information is related to the high-level controller, which is the one running from the ROS planner. Linear speed is represented as the scalar Vx, in m/s, while angular speed is in rad/s, being positive when turning clockwise.

Inside those graphics, we can see the linear and angular speed commands generated by the ROS planner, which are identified as *reference* data lines. Those are the commands sent to the low-level controller, via the IWBus, as represented in Figure 14. Actual vehicle speed data are included over the command lines. Both vehicle behaviors show appropriate performances, taking into account their message-based natures, and they follow the command set-points with reasonable delays and noise. As expected, the KATE platform has higher noise due to the reduced resolution of available motor encoders.

It is possible to identify four similar sectors in each graphic. In the case of the linear speed, we can see clearly when the platform is navigating forwards, along the straight paths from the square sides. Meanwhile, the angular speeds change sharply when the platform has to turn in the corners of the square, including the fourth one when recovering the initial orientation, of 90°, set for the origin point at (0,0).

#### 6.2.2. Indoor Environments and Global Planner

The preferred environments to deploy autonomous robotic wheelchairs are indoor spaces, such as homes, hospitals, commercial areas, or other public buildings [4]. Currently, safety issues of autonomous wheelchairs running outdoors is a question yet to be solved, with legal aspects that need further studies.

When running indoors, having a suitable map of the building is fundamental for global planning algorithms, which are the ones responsible for finding a path between the current position and the desired goal.

**Figure 22:**
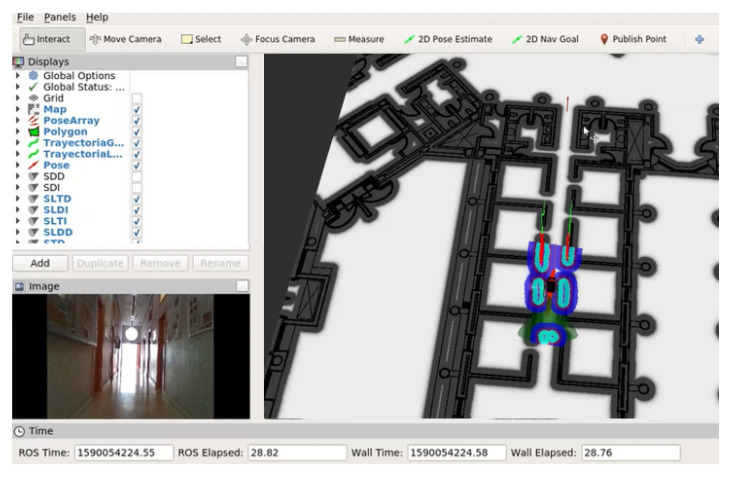
Indoor environment ROS navigation tests. SARA configuration with the 3D camera.

The indoor test environment for SARA and KATE is the Edificio Politecnico of the University of Alcala, which is the location of our working offices and laboratories. Figure 22 shows a snapshot of the robot control application under ROS, where, on the right side, a partial view of the map of the second floor of our building can be seen. The SARA wheelchair is under test, moving along a corridor, using ROS and the 3D camera as the main sensor input device. On the upper left half of the figure, a list of the selected options allows checking the presence of US information coming from the SARA US ring. Finally, in the small window under the list, we can see the RGB output of the 3D camera.

Using this environment, we check the global and local planner behavior of ROS running on our robotic platform. Here, the SARA wheelchair is represented in the middle of the corridor, surrounded by colored shapes that represent the results of the detection of obstacles around the platform. As a reference, we include two short videos, in [57,58], that show the behavior of SARA using a 3D Realsense camera as the primary sensor. In [59], another test is run, but using LiDAR as the primary sensor in this case.

Similar tests were carried out with KATE. The video edition in [60] combines several sources to give a full overview of the moving platform when avoiding a big paper box placed in the middle of the route. In the upper left corner of the video, a partial view of the navigator screen is shown; it is possible to follow the route, the current location of the vehicle, and the localization of the obstacle while in the field of vision of the detection algorithm. In the upper right corner, recordings of the navigation data are shown: the first row, first column shows the 2D path; the second row, first column shows linear and angular command speed sent from ROS to KATE platform; the second column shows right and left actual speeds, derived from encoder readings.

To demonstrate future applications derived from the use of advanced sensors and processors on board an IW, we include here a short video showing preliminary results of a “follow me” behavior, currently under development [61]. Here, we process the images coming from the 3D camera to recognize the gestures of a human located in front of the IW, which will start and stop the mentioned behavior.

### 6.3. Cost Estimation of Materials and Components

Throughout this paper, we mentioned our goal of proposing a low-cost platform for an IW. Cost estimation must take into account not only materials, but also many other aspects related to production: enclosures, boards, mechanical components, industrial profits, distribution costs, homologation procedures, and many others, which are outside the scope of this document. However, we will document here some key concepts that will make clear the advantages of a solution as open as the one proposed here.

Working with standards and open solutions always has a high impact on reducing costs and improving the performance of complex systems [62]. Using these resources helps manufacturers deliver better vehicles, with better features, at reasonable costs to end users. This will also be the case for the designers of different human–machine interfaces, adapted to a wider range of potential users.

Considering only the materials, the components of the KATE platform cost around EUR 480 (year 2022), without the seat (we used a standard children car seat). That budget includes an aluminum platform, wheels, batteries, two DC motors with encoders, motor drivers, joystick and microcontroller components, cabling, and connectors. The adult platform, SARA, was built on a basic electric wheelchair that can now be found for less than EUR 700; together with some additional necessary components (encoders and cabling), this cost increases by about EUR 200.

Depending on the system configuration, different modules can be installed, so the final cost depends on the number and type of such modules. Starting with KATE’s high-level processor (PC node), we installed a Raspberry Pi 4, with a touch screen as input device (around EUR 250). In SARA, the PC node is a laptop with a touch screen, the cost of which ranges from EUR 700 to EUR 1000. However, PC nodes can also be used for other tasks besides IW management (communications, domotics, entertainment, etc.), so their impact on the overall IW cost can be reduced.

Most of the IW performances and applications are based on his sensory system. Those installed in the current sensor node range from EUR 180 for all nine I2C ultrasonic sensors (about EUR 20 each) to the very low price of modern IMU chips, below EUR 5 in any case. Costs for the higher-level sensors used in our work are around EUR 100, for the Slamtec RPLidar A1 [55], and EUR 450, for the Intel Realsense D435 3D camera [54].

Table 3 summarizes the figures given here. Please note that the list covers only components and materials purchased as individual units. On the one hand, OEM prices for electronic components can be much lower; on the other hand, many other costs are not considered here, as mentioned at the beginning of the current section.

## 7. Conclusions

After more than 30 years of active research on the so-called intelligent (or robotic) wheelchairs, the industry has not shown enough interest in this field, so those advancements in research labs cannot reach potential end users—those with strong mobility restrictions.

Nevertheless, the efforts of many research groups around the world continue to be focused on this issue, proposing new applications, systems, and solutions. Bibliographic searches in the main online databases, such as IEEE Xplore or Google Scholar, yield dozens, and even hundreds, of references per year within this research area [3,20,21]. However, most of those references do not take into account the portability of their studies to the market, where a suitable mobile platform must be available.

Our contribution to this topic, within the area of assistive technologies, is the proposal of an open framework for researchers and manufacturers capable of building interoperable systems, which can be interconnected at all levels of abstraction (hardware and software).

The main bottleneck of current systems is access to the physical resources of electric wheelchairs, which is achieved through closed and proprietary interfaces. We proposed an open IWBus, based on a serial bus and a CAN message set, capable of transferring power, dynamic information, and commands between the powered platform and the electronic control modules. Around the IWBus, we tested the feasibility of the system, using standard modules in both hardware and software. The use of standards helps to reduce the cost of the system at all levels: design, materials, development, assembly, maintenance, and documentation.

The authors think that the proposal made here, a layered and modular IW based on standard modules and interfaces, will help with filling the gap between labs and users by reducing costs at each phase of production. The advantages for research laboratories will also be related to the reduction of efforts when adjusting a current electric wheelchair to any specific task or profile.

As a demonstration of the feasibility of the IW concept, on the chassis of a standard electric wheelchair, we built a modular and scalable platform using low-cost electronic modules and a standard serial protocol. This distributed system forms a mobile platform capable of serving a wide range of potential users, from the most basic wheelchair (joystick and motors only) to the most advanced robotic vehicle based on computers, ROS, and high-level sensors.

Different modules were designed and implemented, with different functionalities and complexities, following the IWBus concept. As a result, some modules can be used on either platform with little or no modifications (such as joysticks or sensors), while others can be upgraded or downgraded based on user profiles and specific applications. This is the case of the central processor, which can be an embedded PC (IW adults, SARA) or a single-board computer (SBC, such as a Raspberry Pi, for KATE).

Tests were made on both platforms on several driving modes, from manual direct drive to autonomous navigation using ROS as a high-level controller. Results confirm the validity of the proposal in every situation.

Once a valid platform is available, future actions and future work should be oriented in two very different but complementary directions: legal regulation and research. On the one hand, legal norms are essential to deal with safety issues, insurance companies, and national health services. In this context, the associations of users and caregivers have a fundamental role in bringing the IW to society. On the other hand, further and future research should focus on human–machine interfaces (HMIs) to include users in the IW control loop, because they are by far the best controller for any IW.

Now is the time to answer the question that is often heard at fairs, tests, and exhibitions: Where can I buy this wheelchair?

## Figures and Tables

**Table 1:** Message structure of the CAN bus implemented.

Transmitter	ID	Message A (4 bytes)				Message B (4 bytes)	
Joystick	0x110 (272)	Joystick X		wL	wR	Joystick Y	Mode
	0x111 (273)	Mode (PC)				-	
Motor	0x101 (257)	Encoder R Abs				Time R Abs	
	0x102 (258)	Encoder L Abs				Time L Abs	
	0x210 (528)	Battery level				-	
PC	0x120 (288)	-		wL	wR	Loop	
Sensors	0x201 (513)	SDI		STD		SLDD	SLIT
	0x202 (514)	SLID		SDD		SLDT	STI
	0x203 (515)		Z Axe Acc.	SJO		X Axe Acc.	Y Axe Acc.

**Table 2:** Timing on the CAN bus.

Transmitter	ID	Period	Counts	Variance	Percentage of Occupation
Joystick	0x110 (272)	100.0 ms	3369	0.1 ms	0.118%
Motor	0x101 (257)	100.0 ms	3341	0.3 ms	0.118%
	0x102 (258)	100.0 ms	3341	0.3 ms	0.118%
	0x210 (528)	2000.0 ms	49	0.5 ms	0.0059%
PC	0x120 (288)	100 ms	1023	3.0 ms	0.118%
Sensors	0x201 (513)	200.0 ms	487	0.1 ms	0.059%
	0x202 (514)	200.0 ms	487	0.1 ms	0.059%
	0x203 (515)	200.0 ms	487	0.1 ms	0.059%

**Table 3:** KATE and SARA: cost estimation of materials and components.

Item	EUR
KATE platform: chassis, batteries, motors	EUR 475.00
SARA platform, on a basic electric wheelchair	EUR 900.00
PC node, based on an SBC Raspberry Pi 4	EUR 250.00
PC node, laptop-based (average)	EUR 850.00
Sensor node, 9 US ring + IMU chip	EUR 190.00
Laser rangefinder: Slamtec RPLidar A1	EUR 100.00
3D camera: Intel Realsense D435	EUR 450.00

## Data Availability

No new data were created or analyzed in this study. Data sharing is not applicable to this article.
